# Early Detection of Myeloid-Derived Suppressor Cells in the Lung Pre-Metastatic Niche by Shortwave Infrared Nanoprobes

**DOI:** 10.3390/pharmaceutics16040549

**Published:** 2024-04-17

**Authors:** Jake N. Siebert, Jay V. Shah, Mei Chee Tan, Richard E. Riman, Mark C. Pierce, Edmund C. Lattime, Vidya Ganapathy, Prabhas V. Moghe

**Affiliations:** 1 Department of Biomedical Engineering, Rutgers University, 599 Taylor Rd., Piscataway, NJ 08854, USA; 2 Engineering Product Development, Singapore University of Technology and Design, 8 Somapah Rd., Singapore 487372, Singapore; 3 Department of Materials Science and Engineering, Rutgers University, 607 Taylor Rd., Piscataway, NJ 08854, USA; 4 Rutgers Cancer Institute of New Jersey, 195 Little Albany St., New Brunswick, NJ 08901, USA; 5 Alex’s Lemonade Stand Foundation for Childhood Cancer, 333 E. Lancaster Ave., #414, Wynnewood, PA 19096, USA; 6 Department of Chemical and Biochemical Engineering, Rutgers University, 98 Brett Rd., Piscataway, NJ 08854, USA

**Keywords:** myeloid-derived suppressor cells, shortwave infrared imaging, rare-earth nanoprobes, pre-metastatic niche, prognostication

## Abstract

Metastatic breast cancer remains a significant source of mortality amongst breast cancer patients and is generally considered incurable in part due to the difficulty in detection of early micro-metastases. The pre-metastatic niche (PMN) is a tissue microenvironment that has undergone changes to support the colonization and growth of circulating tumor cells, a key component of which is the myeloid-derived suppressor cell (MDSC). Therefore, the MDSC has been identified as a potential biomarker for PMN formation, the detection of which would enable clinicians to proactively treat metastases. However, there is currently no technology capable of the in situ detection of MDSCs available in the clinic. Here, we propose the use of shortwave infrared-emitting nanoprobes for the tracking of MDSCs and identification of the PMN. Our rare-earth albumin nanocomposites (ReANCs) are engineered to bind the Gr-1 surface marker of murine MDSCs. When delivered intravenously in murine models of breast cancer with high rates of metastasis, the targeted ReANCs demonstrated an increase in localization to the lungs in comparison to control ReANCs. However, no difference was seen in the model with slower rates of metastasis. This highlights the potential utility of MDSC-targeted nanoprobes to assess PMN development and prognosticate disease progression.

## 1. Introduction

Detection and treatment methods have had a profound impact on the survival rates for breast cancer; however, it remains the second leading cause of cancer death amongst women in the United States, with 43,170 deaths predicted in 2023 alone [1]. Approximately 90% of these deaths can be linked directly to distant metastases, and greater than 60% of patients who died were previously diagnosed with a lung metastasis [2,3]. While many patients have de novo metastases at the time of original diagnosis, those with metastatic relapse, or a metastasis-free interval of less than two years, have worse prognostic outcomes, with a median survival of just 12.1 months [4,5]. Recent evidence has also indicated that oligometastatic disease may be indicative of therapy response and an improved prognosis [6,7]. Once metastases have spread to multiple organs and reached tumor sizes greater than 5 cm in diameter, there is a significant decline in survival rates [6,8,9]. Despite significant rates of reoccurrence, distal metastasis screening is not performed in asymptomatic patients [10,11]. Studies in the 1990s failed to demonstrate improved clinical outcomes with screening for metastasis [12,13]; however, these studies were performed with traditional imaging that suffered from poor spatial resolution in contrast to emerging technologies. In contrast, the data supporting the use of adjuvant chemotherapy make it clear that there are survival benefits for the treatment of disease currently considered undetectable [14]. Currently, patients are stratified at the time of initial treatment for long-term adjuvant therapy. Longitudinal monitoring of metastatic risk over this 2-year span may inform clinicians about the need to adjust and tailor appropriate treatment regimens. However, the early monitoring of metastatic disease remains a challenge due to the lack of reliable biomarkers and tools that can provide insight into early changes in the metastatic site.

**Figure 1:**
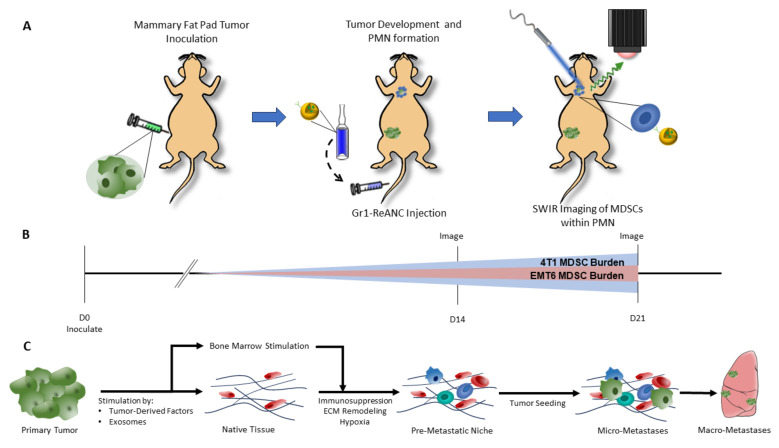
Diagram of shortwave infrared (SWIR) imaging of myeloid-derived suppressor cells (MDSCs) within the lung pre-metastatic niche with rare earth-encapsulated albumin nanocomposites (ReANCs). (**A**) ReANCs engineered with anti-Gr-1 antibodies target MDSCs infiltrating the lung pre-metastatic niche when delivered by tail-vein injection to syngeneic mouse models of breast cancer. (**B**) Study timeline depicting MDSC accumulation over time within the lung pre-metastatic niche, with a greater overall MDSC burden in the 4T1 model reflecting its high metastatic propensity compared to the EMT6 model. SWIR imaging is performed on day 14 and day 21 post-inoculation. (**C**) Diagram of pre-metastatic niche formation. The primary tumor secretes tumor-derived factors and exosomes to stimulate native tissue of the secondary site (lung) and bone marrow. Remodeling of the extracellular matrix, hypoxia, and immunosuppression via the recruitment of MDSCs, regulatory T cells, and macrophages results in the formation of the pre-metastatic nice. This niche is a favorable environment for seeding by circulating tumor cells and metastasis growth.

The development of the pre-metastatic niche (PMN), a microenvironment suitable for colonization by circulating tumor cells as a result of molecular and cellular changes influenced by the primary tumor (Figure 1C), is one of the first changes in metastases [15]. Advances in our understanding of the PMN have established that tumor-secreted factors drive the recruitment of immune cells to the PMN, including T regulatory cells, tumor-associated macrophages, and myeloid-derived suppressor cells (MDSCs). These immune cells prime the microenvironment for tumor cell colonization through immunosuppression, inflammation, and the remodeling of the extracellular matrix [16,17,18]. MDSCs are particularly critical to PMN formation due to their participation in multiple phases of niche development. MDSCs contribute to immune suppression through the direct T-cell inhibition and recruitment of T regulatory cells, drive inflammation through the expression of S100A proteins, and promote tumor colonization by increasing vascular permeability and the induction of E-selectin expression to promote circulating tumor cell arrest and extravasation [17,18]. Due to their key role in PMN formation, and their relative abundance in the lung niche prior to formation of micro-metastases, MDSCs have been identified as a potential biomarker for the PMN [17,19].

Current clinical imaging modalities have proven insufficient for detection of the intricate changes in the PMN. There have been recent attempts to develop S100A-targeted probes for detection of the PMN, using single-positron emission computed tomography (SPECT) [20]. While this is a promising advancement, SPECT and positron emission tomography (PET) are associated with high costs and exposure to ionizing radiation, limiting their utility in longitudinal imaging for the tracking of PMN development [21]. Luminescent nanoparticles targeted to CXCR2 were shown to be effective in tracking the PMN in a mouse model; however, these particles are heavily limited by their peak emission wavelength of 620 nm and have the disadvantage of high tissue scattering and autofluorescence [22,23,24,25,26].

Optical imaging in the near infrared (NIR) range offers key advantages over traditional imaging modalities and imaging in the visible spectrum [27,28]. Optical imaging benefits from high spatial resolution and improved sensitivity, without exposure to ionizing radiation or prolonged scan times [27,29]. We previously designed a rare-earth nanoprobe (RE) that emits in the shortwave infrared (SWIR) region, otherwise known as the second NIR window (NIR-II, 1000–1700 nm) [30]. SWIR light has reduced tissue scattering and tissue autofluorescence compared to alternative optical imaging wavelengths, thus allowing for deeper tissue imaging with high signal-to-noise ratios [28,31]. Additionally, RE nanoprobes are photostable, exhibiting no signs of photobleaching when exposed to continuous excitation in contrast to alternative agents for infrared imaging such as indocyanine green (ICG) [32]. These RE nanoprobes are encapsulated into albumin nanocomposites (ReANCs) for improved biocompatibility and targeting capabilities [33,34,35,36,37]. Previous studies have confirmed that encapsulated ReANCs are non-toxic at high concentrations in vitro and exhibit no signs of in vivo toxicity after repeated injections in mice, including no signs of microscopic changes within organs of clearance [30,34,38]. We previously demonstrated that tumor-targeted ReANCs are able to target and detect micro-metastases in the lungs earlier than magnetic resonance imaging (MRI) and longitudinally track response to therapy [33,34]. Molecularly targeted ReANCs have been shown to be highly specific [36], demonstrating preferential accumulation only in tumors expressing the biomarker corresponding to the targeting ligand when injected into multi-tumor models [34,35,36]. These ReANCs have recently been shown to hold promise for immunosurveillance of CD8^+^ T-cell infiltration within the primary tumor [39].

In this study, we used MDSC-targeted ReANCs to image the lung PMN in a syngeneic orthotopic model of breast cancer with spontaneous metastasis (Figure 1A). We demonstrated that albumin nanocomposites (ANCs) conjugated with anti-Gr-1 antibodies bind mouse MDSCs with high specificity through in vitro assays. We validated that MDSCs are a viable biomarker for metastases by correlating MDSC lung-infiltration to an increased number of metastases in two models of murine breast cancer, 4T1 and EMT6. The MDSC lung infiltration is hypothesized to increase in correspondence to PMN formation over time, with greater increases expected to be seen in the 4T1 model (Figure 1B). Proof-of-concept studies in the two models established target specificity and metastasis tracking potential using MDSC-targeted ReANCs in the lung PMN. In the highly metastatic 4T1 tumor model we found a significant increase in SWIR-associated fluorescence from the MDSC-targeted probes, in contrast to the less aggressive EMT6 model that elicited no or a modest increase in SWIR fluorescence when compared to non-specific probes. We conclude that real-time SWIR imaging with MDSC-targeted ReANCs has the potential to revolutionize the non-invasive early detection of the PMN and prognostication of disease progression, which could be pivotal to improved follow-up care in breast cancer patients.

## 2. Materials and Methods

### 2.1. Rare-Earth Albumin Nanocomposite Synthesis

Rare earth (RE) nanoprobes were synthesized, as previously described, by burst nucleation [30,40]. In brief, yttrium carbonate (0.78 mmol), ytterbium carbonate (0.2 mmol), and erbium carbonate (0.02 mmol) were dissolved together in excess trifluoroacetate to form trifluoroacetate precursors [30,38]. The trifluoroacetate solvent was evaporated by heating the solution at 80 °C until a dried powder was obtained, and the precursors were then dissolved in 50 mL of oleylamine. Then, 25 mL of precursor solution was added to a three-neck round-bottom reaction flask with 2 mmol sodium trifluoroacetate and heated to 100 °C in vacuo over 10 min. Vacuum was stopped, and the solution was rapidly heated to 340 °C under argon for 30 min to form nanoprobes. The undoped NaYF_4_ shell was created around the nanoprobes via the dropwise addition of 1 mmol yttrium trifluoroacetate and 2 mmol sodium trifluoroacetate in 5 mL oleylamine and an additional 30 min of heating before being allowed to cool [30,38]. Final RE nanoprobes are composed of a NaYF_4_ core doped with ytterbium (Yb) and erbium (Er) and an undoped NaYF_4_ shell. RE nanoprobes were purified via 3 cycles of centrifugation at 48,400× *g* and resuspension in isopropyl alcohol to remove residual oleylamine; the final resuspension was performed in water, flash frozen in liquid nitrogen, and lyophilized to form a powder used for ReANC synthesis. Complete UV/Vis/IR spectra of the RE nanoprobes were previously published by Naczynski et al. (2013) [30].

Synthesis of albumin nanocomposites (ANCs) and rare-earth albumin nanocomposites (ReANCs) via solvent-induced controlled coacervation was performed as previously reported [30,34,35]. In brief, human serum albumin (HSA) (Millipore Sigma, Burlington, MA, USA) was dissolved in 10 mM NaCl at 20 mg mL^−1^ and adjusted to a pH of 8.50 ± 0.05 with 0.1 M NaOH. RE nanoprobes were dissolved at 0.4 mg mL^−1^ in 100% ethanol, using a bath ultrasonicator for 1 h. For ANC synthesis, 100% ethanol was used without RE nanoprobes. To incorporate fluorescent dyes for in vitro and flow cytometry experiments, fluorescein isothiocyanate (FITC) was added to the ethanol solution at 12.6 µg mL^−1^. A syringe pump was used to infuse 2 mL of the ethanol solution to 500 µL of HSA solution in a 20 mL scintillation vial, with continuous stirring at 1000 RPM on a magnetic stirrer. After infusion, 2.34 µL of 8% glutaraldehyde was added to the vial, which was then covered and allowed to stir for 16–18 h. Purification of nanocomposites was performed through two rounds of high-speed centrifugation (Avanti J-E Centrifuge, Beckman Coulter, Brea, CA, USA) at 48,000× *g* for 10 min at 4 °C and resuspended in phosphate-buffered saline (PBS) at 5 mg mL^−1^.

### 2.2. Nanocomposite Functionalization

The ANCs and ReANCs were re-suspended in PBS + 1 mM ethylenediaminetetraacetic acid (EDTA) following the second round of centrifugation. Free amines on the surface of the albumin shell were then thiolated using Traut’s reagent (2-iminothiolane) [41]. Traut’s reagent (Thermo Fisher Scientific, Waltham, MA, USA) was dissolved in water at a concentration of 2 mg mL^−1^ and added to nanocomposite suspension with 2 mg of Traut’s reagent per 17.5 mg HSA. The solution was placed on a shaker for 1 h at 600 RPM prior to purification at 48,000× *g* for 10 min at 4 °C and resuspended in PBS + 1 mM EDTA at 5 mg mL^−1^ of HSA.

Immunoglobulin G (IgG) antibodies were conjugated to the thiol groups added to the albumin shell through maleimide chemistry [41]. Sulfosuccinimidyl 4-(N-maleimidomethyl)cyclohexane-1-carboxylate (sulfo-SMCC) (Thermo Fisher Scientific, Waltham, MA, USA) was dissolved in water at 1 mg mL^−1^. Anti-mouse Gr-1 rat IgG2b antibodies (clone NIMP-R14, Bio X Cell, Lebanon, NH, USA) or isotype control rat anti-trinitrophenol IgG2a antibodies (clone 2A3, Bio X Cell) were prepared in PBS + 1 mM EDTA. The sulfo-SMCC solution was added to the IgG solution at a 10-fold molar excess. This mixture was then reacted for 30 min at room temperature. Maleimide-activated IgG was purified using an Amicon Ultra centrifugal filter with a 10,000 kDa molecular weight cutoff (Millipore Sigma, Burlington, MA, USA), centrifuged at 14,000× *g* for 15 min. IgG was resuspended in PBS + 1 mM EDTA, and 500 µL of maleimide-activated IgG was added to 3.5 mL of nanocomposites. For ANCs used during in vitro experiments, the IgG concentration was varied from 3.4 µg IgG per mg HSA up to 42.9 µg IgG per mg HSA. For in vivo experiments, activated IgG was added to ReANCs at a ratio of 28.6 µg IgG per mg HSA. Following the addition of activated IgG to the nanocomposites, the solution was mixed for 30 min on a shaker at 600 RPM. The conjugated nanocomposites were purified via centrifugation at 48,400× *g* for 10 min at 4 °C and resuspended in PBS at a concentration of 50 mg HSA mL^−1^.

### 2.3. Nanocomposite Characterization

The yield of nanocomposites was determined by bicinchoninic acid (BCA) assay (Thermo Fisher Scientific, Waltham, MA, USA) of the purification supernatants after synthesis to estimate total protein content. Nanocomposite size and polydispersity were determined by dynamic light scattering (DLS) in a DynaPro^®^ plate reader (Wyatt Technology, Santa Barbara, CA, USA).

IgG antibody loading was quantified by a direct enzyme-linked immunosorbent assay (ELISA), as described previously [39]. In brief, ReANCs were diluted to a maximal conjugated IgG concentration of 35.7 µg mL^−1^, and 100 µL of the diluted particles was incubated on Nunc™ Maxisorp 96-well plates (Thermo Fisher Scientific, Waltham, MA, USA) at 4 °C, overnight. Wells were then washed with PBS + 0.05% Tween and blocked with 1% bovine serum albumin (BSA) in PBS for 1 h. Wells were then incubated with goat anti-rat IgG H&L conjugated with horseradish peroxidase (HRP) (ab205720, Abcam, Waltham, MA, USA) in 1% BSA solution for 2 h. Next, the wells were washed with PBS + 0.05% Tween prior to incubation with 100 µL tetramethylbenzidine (TMB) substrate (R&D Systems, Minneapolis, MN, USA) for 20 min; the reaction was stopped with 50 µL of 2 N sulfuric acid. The light absorbance was read at 450 nm with reference to 570 nm on a plate reader (Tecan, Männedorf, Switzerland). The OD from a standard curve of antibody was compared against the OD of the samples to calculate antibody conjugation efficiency.

### 2.4. Cell Culture

4T1 (American Type Culture Collection [ATCC], Old Town Manassas, VA, USA) mouse mammary cancer cells were cultured in Dulbecco’s Modified Eagle Medium (DMEM) supplemented with 10% fetal bovine serum (FBS) and 1% penicillin/streptomycin. EMT6 (ATCC, Old Town Manassas, VA, USA) mouse mammary cancer cells were cultured in Waymouth’s MB 752/1 media supplemented with 10% FBS serum and 1% penicillin/streptomycin. Cells were incubated at 37 °C and 5% CO_2_.

### 2.5. Animals

All studies performed in this report involving animals were approved triennially in March 2022 by the Institutional Animal Care and Use Committee (IACUC) of Rutgers University under project “ID999900469—Nanophotonic imaging of tumors”. Research was performed in accordance with institutional guidelines on animal handling. Athymic and Balb/c mice were purchased from Charles River Laboratories and received at 6 weeks of age. They were given a minimum of 48 h to acclimate prior to the initiation of the study. Mice were provided with food and water ad libitum.

### 2.6. MDSC Isolation

Spleens were resected from 4T1 tumor-bearing mice on day 10–20 post-inoculation. Spleens were manually crushed and filtered through a 40 µm cell strainer to obtain a single cell suspension. Cells were resuspended in PBS + 2% FBS + 1 mM EDTA at a concentration of 1 × 10^8^ cells mL^−1^ prior to negative selection of MDSCs with the EasySep™ Mouse MDSC (CD11b^+^Gr1^+^) Isolation Kit (Stemcell Technologies, Vancouver, BC, Canada) according to manufacturer instructions.

### 2.7. Nanocomposite Targeting Assessment by Flow Cytometry

Isolated mouse MDSCs were resuspended in RPMI 1640 media supplemented with 10% FBS and 1% penicillin/streptomycin, and 1 × 10^5^ cells were plated into a U-bottom 96-well plate. MDSCs were treated with 10 µL of FITC-labeled ANCs (0.5 mg HSA mL^−1^) with varying loading concentrations of either anti-Gr-1 or isotype control antibodies. Cells were incubated with ANCs for 1 h at room temperature, with shaking, and then centrifuged and washed with cold PBS three times. Cells were fixed with 1% PFA for 15 min at room temperature and re-suspended in PBS. Binding was analyzed on a Gallios flow cytometer (Beckman Coulter). ANC binding was assessed by FITC intensity. All samples were run with three technical replicates and repeated in three experiments. Statistical differences were determined using a one-way analysis of variance (ANOVA) with a Tukey’s post hoc test for multiple comparisons.

### 2.8. Tumor Models

Following acclimatization periods, mice were inoculated with either 4T1 or EMT6 cell lines. Mice were anesthetized in 2–3% isoflurane (Henry Schein, Melville, NY, USA). For Balb/c mice, hair was removed from the area around the 4th right teat with Nair™ hair removal cream, and the skin was thoroughly cleaned. Inoculation was performed by injecting 2 × 10^4^ cells into the mammary fat pad under the 4th right teat with a 30G ½ inch syringe [42]. Tumors were monitored daily and measured three times a week. Tumor volume was calculated using Equation (1), where *l* is the longest dimension of the tumor, and *w* is the perpendicular dimension.

(1) $$V=\frac{l\times w^{2}}{2}$$

Mice were monitored for humane endpoints, including tumor ulceration, declining body condition score, or >10% weight loss. Mice meeting criteria for humane endpoints were immediately euthanized.

### 2.9. ReANC Administration and SWIR Imaging

SWIR imaging was performed on days 14 and 21 post-cell inoculation in Balb/c mice (Figure 1A,B) and days 12 and 17 in athymic mice. One day prior to ReANC injections, pre-injection SWIR imaging was performed. Balb/c mice had hair over the chest and abdomen removed using Nair™ hair removal cream. Imaging was performed using a custom small-animal SWIR imaging system, as previously described [33,34,35,36,39]. On the day of imaging, mice received 100 µL of either Gr-ReANC or Iso-ReANC (50 mg HSA mL^−1^) intravenously through the tail vein with a 27G ½” syringe. After 6 h, SWIR imaging was repeated to capture ReANC signal. During imaging, mice were anesthetized with 2–3% isoflurane and scanned with a continuous-wave 980 nm collimated laser (12 mm beam diameter) with a power output of 1.7 W. Rare-earth emissions were captured by a 512 × 640-pixel InGaAs camera (640HSX, Sensors Unlimited, Princeton, NJ, USA). The camera was fitted with two 1150 nm long-pass filters (Semrock, Rochester, NY, USA), a 1497–1579 nm band-pass filter (Semrock), and a 25 mm fixed focal length f/1.4 SWIR lens (StingRay Optics, Keene, NH, USA) to capture wavelengths within the ReANC SWIR emission spectrum. Videos were captured at 30 frames per second (33 ms exposure time) and stored as .bin files as the illumination beam was scanned; these were converted to .tiff image files using a custom MATLAB (version R2020a Update 5 [9.8.0.1451342]) script to capture the maximum intensity values for each pixel across all frames of the video. A white-light image was captured prior to SWIR imaging with 1550 nm LED illumination and used for anatomical reference and SWIR signal overlays [35]. Ex vivo SWIR imaging was performed by resecting tissues from mice euthanized by CO_2_ inhalation 6–8 h post-ReANC injection. Organs were placed on a non-reflective mat and scanned with the 980 nm laser to capture SWIR emissions, as described above.

### 2.10. Image Analysis

Image analysis was performed using FIJI open-source software (version 1.54f). Regions of interest (ROIs) were drawn around anatomical reference points on the white-light image. Quantification was performed by taking the average pixel intensity in the ROI of the 6-h timepoint image and subtracting out the mean pixel intensity of the same ROI in the pre-injection image. To account for variation in ReANC emission intensity between experiments performed in Balb/c mice, data are presented as study averages with a ratio paired *t*-test between the study averages of Gr-ReANC and Iso-ReANC values. Data from the study of athymic mice are presented as individual values.

### 2.11. Metastasis Counting

Lungs from mice on day 21–28 post-inoculation were resected, rinsed with PBS, and stored in 10% formalin for 24–48 h prior to storage in 75% EtOH. Fixed lungs were analyzed under a dissecting microscope for evidence of metastasis, defined as raised bumps and pale discolorations in the healthy pink lung tissue. Metastases were counted and analyzed with Student’s *t*-test.

### 2.12. Immunohistochemistry

Tissue samples from Balb/c mice were resected and fixed in 10% formalin for 24–48 h and then transferred to 75% EtOH and stored at 4 °C. Tissues were paraffin-embedded and cut with a microtome into 5 µm slices for mounting on glass slides. Slides were then de-paraffinized with xylene and re-hydrated with EtOH dilutions. Antigen retrieval was performed with boiling citrate buffer for 20 min. Slides were washed with tris-buffered saline (TBS) and blocked with Protein Block (ab64226, abcam) overnight at 4 °C. Slides were then incubated with primary antibodies diluted in TBS + 2.5% BSA + 10% goat serum for 1 h at room temperature or overnight at 4 °C. Rat anti-mouse Gr-1 (clone NIMP-R14, Bio X Cell) was diluted to 1 µg/mL, polyclonal rabbit anti-mouse CD11b (PA5-79533, Thermo Fisher Scientific, Waltham, MA, USA) was diluted to 0.5 µg/mL, and monoclonal rabbit anti-mouse Ki-67 antibodies (clone D3B5, Cell Signaling Technology, Danvers, MA, USA) were diluted 1:1000. Slides were washed with TBS, and endogenous peroxidase was blocked with 3% hydrogen peroxide for 10 min at room temperature, followed by TBS washes to remove excess hydrogen peroxide. Secondary antibodies were diluted in TBS + 2.5% BSA + 10% goat serum and added to the slide for 1 h at room temperature. HRP-conjugated secondary antibodies, goat anti-rat (31470, Thermo Fisher Scientific, Waltham, MA, USA) and goat anti-rabbit (ab97051, abcam), were diluted at a concentration of 1:500. Secondary antibody was washed off with TBS, and then slides were incubated with 3,3′-diaminobenzidine (DAB) substrate (Vector Laboratories, Newark, CA, USA) according to manufacturer’s instructions. Slides were washed with water and counterstained for 20–30 s with Mayer’s hematoxylin solution (Millipore Sigma, Burlington, MA, USA) prior to a final wash in water. Slides were dehydrated in 99% isopropanol and cover slipped with VectaMount^®^ permanent mounting medium (Vector Laboratories). Hematoxylin and eosin staining was also performed on tumor, lung, spleen, and liver samples.

### 2.13. Statistical Analysis

All statistical analyses were performed in GraphPad Prism software (version 10.2.1). All data are presented as the mean ± standard error of the mean for bar graphs and dot plots. Violin plots display data distribution as a function of plot width, with dashed lines representing the median and dotted lines for the 25th and 75th percentile. In vivo SWIR imaging results were first averaged by study and then analyzed using a ratio paired *t*-test with a 95% confidence interval to detect differences between Iso-ReANC and Gr-ReANC groups across all studies. The in vitro and ex vivo results were compared using Student’s *t*-test to assess for differences between two groups after removal of outliers. A one-way analysis of variance (ANOVA) was performed to detect differences across multiple groups, with Tukey’s post hoc test to perform multiple comparisons.

## 3. Results

### 3.1. Characterization of MDSC-Targeted Nanocomposites

**Figure 2:**
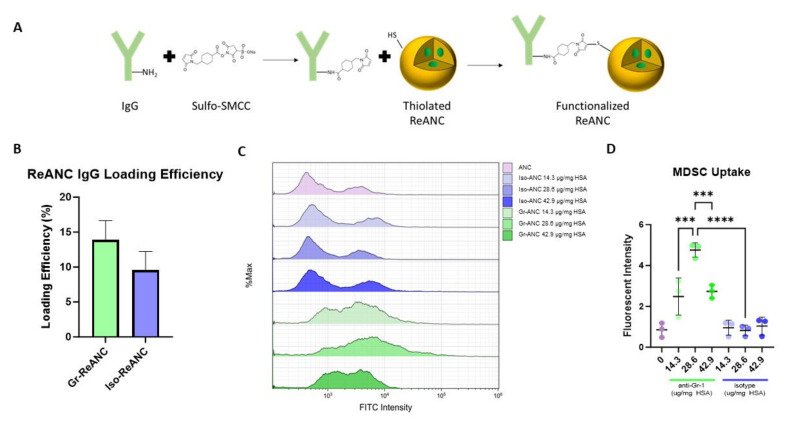
Engineering ReANCs for MDSC targeting by chemical conjugation of antibodies to the albumin shell. (**A**) A schematic illustrating the conjugation of IgG antibodies to the albumin shell of ReANCs. Sulfo-SMCC is used to create a maleimide-activated IgG intermediate, which reacts with sulfhydryl groups on the surface of thiolated nanocomposites. (**B**) Loading efficiency of IgG conjugation to ReANCs, as evaluated by direct ELISA, with a starting concentration of 28.6 µg mg^−1^ human serum albumin (HSA). Average loading efficiencies were 13.9% and 9.6% for Gr-ReANCs and Iso-ReANCs, respectively, with no statistical significance (*n* = 5). (**C**,**D**) Flow cytometry analysis of in vitro MDSC uptake of IgG-conjugated albumin nanocomposites (ANCs) at varying loading densities demonstrates the increased binding of Gr-ANCs than Iso-ANCs. (**C**) FITC fluorescence intensity histograms show increased binding of Gr-ReANCs. No difference in histogram is observed between unconjugated ANCs and Iso-ANCs at any concentration. (**D**) Dot plot demonstrating increased fluorescent intensity among Gr-ANCs compared to ANCs (*p* < 0.0001) and Iso-ANCs (*p* < 0.0001). Peak binding was observed at 28.6 µg anti-Gr-1 mg^−1^ HSA compared to 14.3 (*p* < 0.0001) and 42.9 µg anti-Gr-1 mg^−1^ HSA (*p* = 0.0003). No difference was observed between ANCs and Iso-ANCs (*p* > 0.9999). *** indicates *p* < 0.001; **** indicates *p* < 0.0001.

For MDSC targeting, nanocomposites were functionalized with anti-Gr-1 (anti-Ly6G/Ly6C) immunoglobulin G (IgG) antibodies to form Gr-ANCs or Gr-ReANC and isotype control IgG antibodies for Iso-ANC or Iso-ReANC (Figure 2A). The average loading efficiency assessed by ELISA for Gr-ReANCs is 13.9% and 9.6% for Iso-ReANCs when synthesized with 28.6 µg IgG/mg albumin. The average hydrodynamic diameter of Gr-ReANCs used in this study was 155.0 ± 8.0 nm, and the average diameter of Iso-ReANCs was 153.0 ± 6.7 nm (Supplementary Figure S1A), with polydispersity indices (PDIs) of 0.17 and 0.15, respectively, suggesting that the ReANCs were monodisperse and of equivalent sizes. We did not observe significant differences in loading efficiencies between the two antibodies (Figure 2B, *n* = 5) or the size and PDI of the ReANCs.

### 3.2. Validation of MDSC Targeting

To validate the MDSC targeting capabilities of anti-Gr-1-conjugated nanocomposites, in vitro cell-binding studies were performed. All in vitro binding assays were performed with ANCs, as previous studies have shown that the rare-earth core in ReANCs does not affect the cell binding of the nanocomposite [38]. ANCs labeled with fluorescein isothiocyanate (FITC) were synthesized and functionalized with varying concentrations of antibody (3.6–42.9 µg IgG/mg albumin). MDSCs treated with these functionalized ANCs demonstrated that Gr-ANC-treated cells had greater fluorescent intensity compared to Iso-ANC- and ANC-treated cells regardless of antibody loading density (Figure 2C,D). The peak cell binding of Gr-ANCs occurred when loaded with 28.6 µg anti-Gr-1 IgG/mg albumin and was the density used for the in vivo studies.

### 3.3. Balb/c Breast Cancer Tumor Models

**Figure 3:**
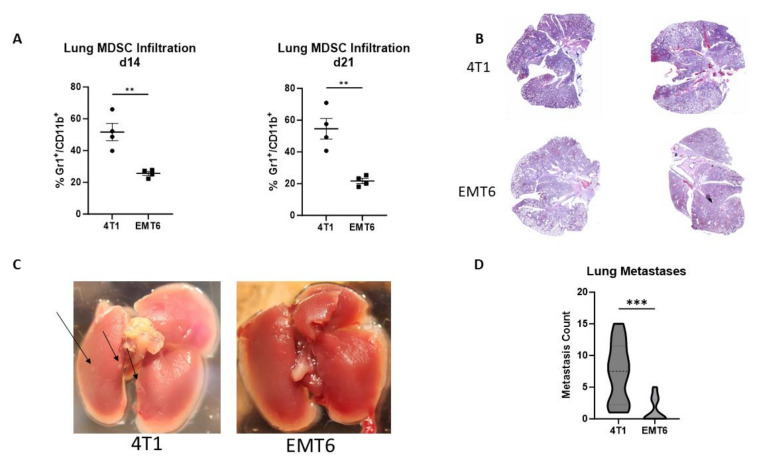
Characterization of spontaneous lung metastasis and MDSC infiltration in two syngeneic orthotopic models of breast cancer, 4T1 and EMT6. (**A**) Flow cytometry analysis of Gr-1^+^/CD11b^+^ MDSCs within the lungs of 4T1 tumor-bearing mice shows a significant elevation on day 14 (*p* = 0.0034) and day 21 (*p* = 0.0026) post-inoculation compared to EMT6 tumor-bearing mice. (**B**) Representative lung H&E images on day 21 post-inoculation. (**C**) Representative lung images on day 21 post-inoculation, with arrows pointing to areas of visible metastasis formation. (**D**) Violin plots of lung metastases quantification; 4T1 mice had increased metastases compared EMT6 mice (*p* = 0.0004). ** indicates *p* < 0.01; *** indicates *p* < 0.001.

The primary models utilized here are Balb/c mice inoculated with the 4T1 or EMT6 cell lines. Previous publications have characterized the 4T1 tumor cell line as having greater metastatic propensity in orthotopic models in comparison to the EMT6 cell line [19,43]. Here, we validate that MDSC lung infiltration is elevated in the model with a greater rate of metastasis. Mice with 4T1 tumors had significantly higher MDSC lung infiltration on day 14 post-inoculation, with 51.66% Gr1^+^/CD11b^+^; and day 21, 54.59%, compared to mice with EMT6 tumors, with 25.61% and 21.72%, respectively (Figure 3A). Ex vivo examination of the lungs on day 28 post-inoculation revealed 4T1 tumor-bearing mice to have an increased number of metastatic lung nodules compared to EMT6 mice. This is reflected in the H&E staining of the lungs, demonstrating decreased normal lung architecture in 4T1 mice (Figure 3B,C). Nodule counting under a dissecting microscope revealed an average of 7.33 metastases in the lungs of 4T1 mice, significantly greater than the average of 1.33 metastases observed in EMT6 tumor-bearing mice (Figure 3D). The immunohistochemistry staining of the lungs demonstrates that mice with 4T1 tumors exhibit increased expression of MDSC markers Gr-1 and CD11b and proliferation marker Ki-67 (Supplementary Figure S5).

### 3.4. Proof-of-Concept Imaging in Athymic Mice

**Figure 4:**
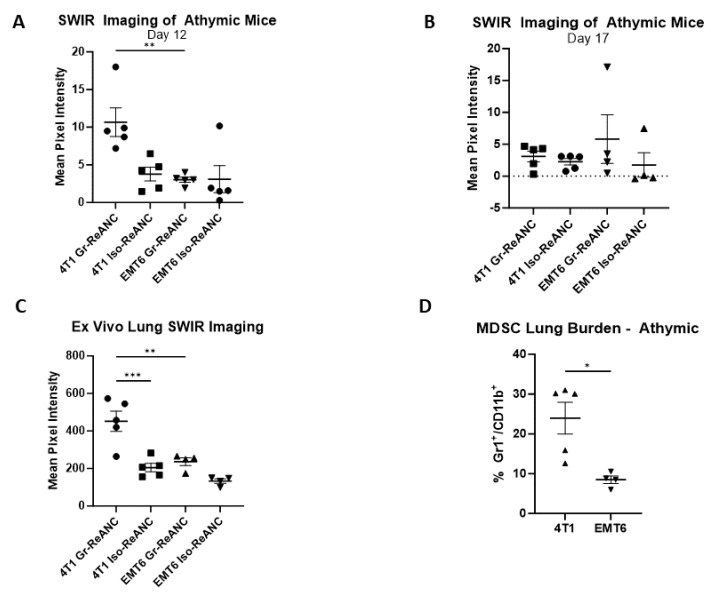
In vivo SWIR imaging of the athymic mouse lungs in 4T1 and EMT6 murine models of breast cancer receiving MDSC-targeted ReANCs. (**A**) Imaging on day 12 post-inoculation revealed increased lung SWIR signal in 4T1 tumor-bearing mice receiving Gr-ReANCs compared to Iso-ReANCs (*p* = 0.009) and EMT6 tumor-bearing mice with Gr-ReANC (*p* = 0.004). No difference was observed between Gr-ReANCs and Iso-ReANCs in EMT6 tumor-bearing mice (*p* > 0.9999). SWIR images are available in Supplementary Figure S8. (**B**) On day 17 post-inoculation, there was no statistical difference observed between any group by ANOVA (*p* = 0.5180). (**C**) Elevated SWIR signal on ex vivo imaging of lungs in 4T1 mice receiving Gr-ReANCs compared to Iso-ReANCs (*p* = 0.0003) and EMT6 mice receiving Gr-ReANC (*p* = 0.0020). No difference was observed between EMT6 Gr-ReANC and EMT6 Iso-ReANC lungs (*p* = 0.1893). (**D**) Flow cytometry analysis of Gr-1^+^/CD11b^+^ cells in the lungs of 4T1 tumor-bearing mice and EMT6 tumor-bearing mice indicates an elevated infiltration of MDSCs in mice with 4T1 tumors (*p* = 0.0164). * indicates *p* < 0.05; ** indicates *p* < 0.01; *** indicates *p* < 0.001.

Due to unavoidable imaging artifacts caused by the presence of hair on immunocompetent mice and consequent signal attenuation, an initial proof-of-concept imaging study was conducted in athymic mice to validate the initial hypothesis, namely that Gr-ReANCs can detect MDSC lung infiltration into the PMN, with minimal imaging artifacts [44]. Mice were inoculated in the mammary fat pad on day 0 with one of two syngeneic murine tumor cell lines: 4T1 or EMT6. On day 12 and day 17, mice received tail vein injections of either Gr-ReANC or Iso-ReANC (*n* = 5/group). At 6 h post-injection on day 12, 4T1 mice receiving Gr-ReANCs had a mean SWIR intensity of 10.66 mean pixel intensity (m.p.i.), significantly greater than the SWIR intensity for 4T1 mice receiving Iso-ReANC (3.78 m.p.i.; *p*-value: 0.006) and for EMT6 mice with Gr-ReANCs (3.03 m.p.i., *p*-value: 0.004), as shown in Figure 4A and Supplementary Figure S8. There was no difference between EMT6 mice receiving Gr-ReANC and Iso-ReANC (3.09 m.p.i.). In Figure 4B it is shown that, on day 17 post-inoculation, there was a non-significant increase in SWIR intensity in mice that received Gr-ReANC (4T1, 3.08 m.p.i.; EMT6, 5.82 m.p.i.) and Iso-ReANC (4T1, 2.25 m.p.i.; EMT6, 1.74 m.p.i.). Ex vivo imaging of the lungs on day 17 revealed a significant increase in 4T1 Gr-ReANC mice (452.0 m.p.i.) compared to both 4T1 Iso-ReANC (205.2 m.p.i.; *p*-value = 0.0003) and EMT6 Gr-ReANC (236.3 m.p.i.; *p*-value = 0.0020), but no significant difference between EMT6 mice that received different ReANC formulations (Figure 4C). The MDSC lung infiltration trends in Balb/c were reflected in the athymic model, where 4T1 mice have significantly increased MDSC lung burden (23.94%) compared to EMT6 mice (8.48%, *p*-value: 0.016) based on flow cytometry from animals that received no ReANC injections (Figure 4D).

### 3.5. In Vivo Imaging of the Pre-Metastatic Niche

**Figure 5:**
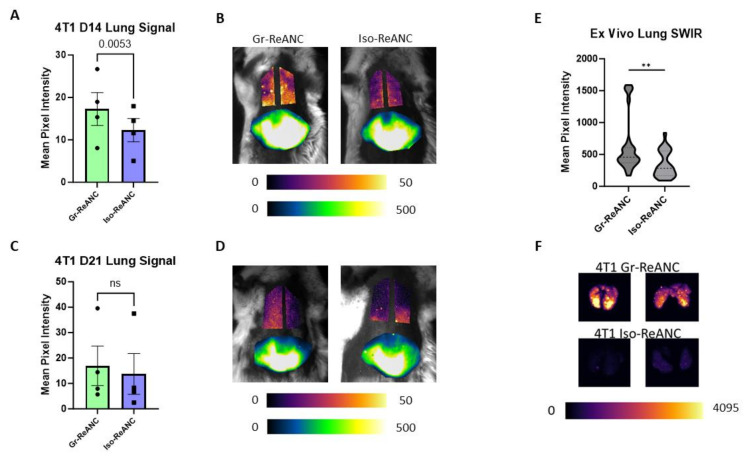
In vivo and ex vivo SWIR imaging of lungs in the 4T1 model of breast cancer. (**A**) Bar graphs of average lung SWIR signal on day 14 post-inoculation, demonstrating increased signal in mice receiving Gr-ReANCs with a paired-ratio *t*-test (*p* = 0.0053); data points are individual study averages (plots of individual studies are shown in Supplementary Figure S6). (**B**) Representative SWIR images on day 14, lung fields are plotted from 0-pixel to 50-pixel intensity and liver from 0-pixel to 500-pixel intensity. (**C**) Average lung SWIR signal on day 21, no significance found between Gr-ReANC and Iso-ReANC with a ratio paired *t*-test (*p* = 0.2662). (**D**) Representative in vivo SWIR images on day 21 in 4T1 mice. (**E**) Violin plot of quantified ex vivo SWIR signal. Average strength of SWIR response is higher in lungs with Gr-ReANCs compared to Iso-ReANCs (*p* = 0.0033). (**F**) Representative ex vivo SWIR images from day 21 imaging. ns = not significant; ** indicates *p* < 0.01.

Next, we evaluated the capabilities of Gr-ReANCs to detect MDSC lung infiltration through in vivo SWIR imaging in two syngeneic orthotopic models of breast cancer. As hypothesized in the athymic mouse model, we expect that the increased MDSC burden will lead to increased accumulation of Gr-ReANCs compared to Iso-ReANCs in mice with 4T1 tumors. On day 14 and day 21 post-inoculation, Balb/c mice received Gr-ReANC or Iso-ReANC tail-vein injections. The lung SWIR imaging of 4T1 mice across four independent studies had an average intensity of 17.27 mean pixel intensity (m.p.i.) for Gr-ReANCs, significantly higher than the average of 12.26 m.p.i. for Iso-ReANCs (Figure 5A,B; *p*-value = 0.0053). On day 21, there was a non-significant increase in SWIR intensity for Gr-ReANC (16.90 m.p.i.) compared to Iso-ReANC (13.76 m.p.i.) (Figure 5C,D). Individual study data can be found in Supplementary Figure S6, demonstrating a consistent increase in average SWIR intensity for Gr-ReANC on day 14 in all studies. Ex vivo imaging of the lungs demonstrates considerably increased SWIR signal, with mice receiving Gr-ReANCs, with a 287.7 m.p.i. difference (Figure 5E; *p*-value = 0.0033).

**Figure 6:**
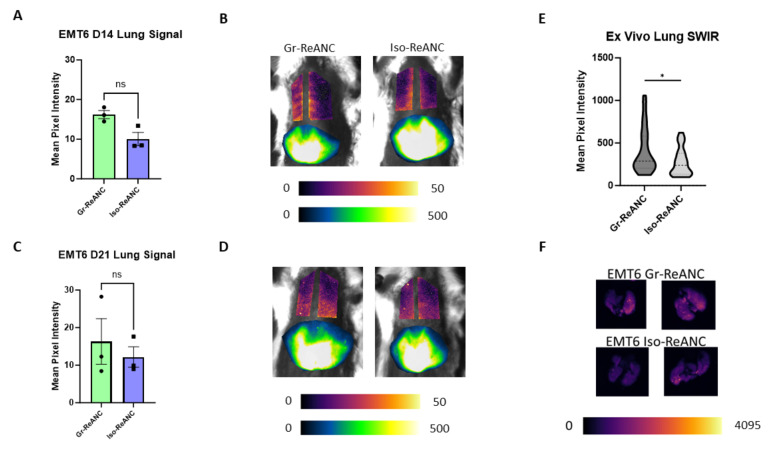
Lung SWIR imaging results in the EMT6 model of breast cancer. (**A**) Average lung signal across three studies on day 21, with no difference between Gr-ReANC and Iso-ReANC groups (*p* = 0.1013). Plotted points represent study averages; individual studies are plotted in Supplementary Figure S7. EMT6 tumor data from 1 study were excluded due to the failure of the tumors to develop. (**B**) Representative SWIR images, with lung fields plotted with pixel intensities 0–50 and livers 0–500. (**C**) Day 21 SWIR imaging of EMT6 mice, no difference in lung signal is detected (*p* = 0.3093). (**D**) Representative SWIR images from day 21. (**E**) Quantification of ex vivo SWIR signal in EMT6 mouse lungs. Gr-ReANC had significantly increased signal compared to Iso-ReANC (*p* = 0.0498). (**F**) Representative lung ex vivo SWIR images of EMT6 tumor-bearing mice. ns = not significant; * indicates *p* < 0.05.

In contrast to 4T1 mice, mice with EMT6 tumors showed no difference across SWIR intensity between Gr-ReANC and Iso-ReANC on both day 14 and day 21, using a comparison with a paired *t*-test. The average SWIR intensity across three studies for Gr-ReANCs was 16.20 m.p.i., compared to 10.05 m.p.i. for Iso-ReANCs, on day 14, as shown in Figure 6A,B. On day 17, the average signal intensity for Gr-ReANC was 16.32 m.p.i., and it was 12.16 m.p.i. for Iso-ReANC (Figure 6C,D). Individual study data are presented in Supplementary Figure S7. However, ex vivo imaging discerns an average difference in lung SWIR intensity in EMT6 tumor-bearing mice, with an average increase of 131.6 m.p.i. in mice receiving Gr-ReANCs (Figure 6E,F).

## 4. Discussion

High rates of distant metastasis, poor prognosis following metastasis, and high rates of recurrence in triple-negative breast cancer resulted in aggressive adjuvant chemotherapy following the removal of the primary tumor [45,46]. Current screening for adjuvant therapy relies on pathological complete response (pCR) in the breast and lymph nodes, with those not achieving pCR receiving adjuvant chemotherapy to reduce the risk of metastasis [47]. Patients and clinicians could benefit from longitudinal methods of assessing metastatic risk, as 15% of patients with pCR and 50% of patients without pCR have recurrent disease within 10 years [47]. Labeling the key components of the pre-metastatic niche (PMN) will allow for longitudinal tracking of metastatic risk, allowing clinicians to better tailor care and adjuvant chemotherapies to the individual patient. The goal of this study was to develop nanoprobes for tracking the development of the PMN as a potential screening tool for breast cancer metastasis. To this end, we investigated whether MDSC-targeted optical nanoprobes can detect the immune dynamics of the PMN with two pre-clinical models of breast cancer that differ in metastatic propensity (see Figure 1) [48].

We first developed a Gr-1 (Ly6G/Ly6C)-targeted nanoparticle through the conjugation of an anti-Gr-1 IgG antibody to the albumin shell of the nanoprobe, using the crosslinker sulfo-SMCC. The Gr-1 (Ly6G/Ly6C) surface marker was chosen as the murine MDSC that is primarily identified by the co-expression of Gr-1 and CD11b [49]. The optimal antibody loading concentration of 28.6 µg IgG mg^−1^ HSA was determined through flow cytometry studies with isolated MDSCs over a range from 3.6 to 42.9 µg IgG mg^−1^ HSA (see Figure 2C and Supplementary Figure S1B). The low binding affinity of control and isotype-conjugated ANCs confirms that non-specific uptake is insignificant compared to the high binding affinity of anti-Gr-1-conjugated ANCs for MDSCs. The decreased MDSC binding at 42.9 µg IgG mg^−1^ HSA compared to 28.6 µg IgG mg^−1^ HSA could be a result of surface Gr-1 sequestration by excess antibody density on the surface. The binding of multiple receptors by a single Gr-ANC will reduce the number of sites available for additional Gr-ANC binding to a single cell. The reduction in Gr-ANC surface density will have a corresponding reduction in signal strength.

Mouse hair is known to attenuate NIR light, resulting in imaging artifacts with artificially reduced SWIR signal in mouse strains with dense hair, such as the Balb/c mice [44]. While hair removal will improve the signal, residual hairs will continue to attenuate the signal, and small abrasions from hair removal create artifacts with elevated signal. Therefore, athymic nude mice were utilized for the initial hypothesis testing to provide an artifact-free proof-of-concept imaging model. This study clearly demonstrates the ability of these SWIR nanoprobes to identify increasing MDSC populations within the lung niche in the 4T1 model with high metastatic potential compared to the EMT6 model with low metastatic propensity (see Figure 4) [19]. The increasing SWIR signal aligns in the 4T1 tumor-bearing mice with the increased number of MDSCs observed within the lungs compared to mice with EMT6 tumors. Interestingly, on Day 17 of the study, there was no difference in the SWIR signal between the two groups. This is hypothesized to be due to enhanced permeability and retention through the leaky vasculature as the PMN develops, reducing the specificity of targeted nanoprobes [50,51].

Ex vivo imaging supports the ability of the nanoprobes to detect the MDSC burden as a function of SWIR fluorescence intensity in the lungs of 4T1 tumor-bearing mice. We detected a significant increase in 4T1 mice that received Gr-ReANCs compared to Iso-ReANCs. Furthermore, in the EMT6 model, as expected, the SWIR intensity was significantly decreased compared to 4T1 mice that received Gr-ReANCs (Figure 4C). However, there was no discernible difference in SWIR fluorescence intensity between Gr-ReANC- and Iso-ReANC-administered mice in EMT6 mice. This is validated by the MDSC burden, as determined in Figure 4D, which demonstrates significantly greater MDSC infiltration in the lungs of 4T1 mice, compared to EMT6.

The athymic model is limited in utility for immune-tracking studies, as it lacks T cells, major players in the PMN (regulatory T cells) and anti-tumor response (cytotoxic T cells). To address these limitations, a total of four independent studies were performed to provide complementary insights, with the utilization of an immunocompetent Balb/c model. These results highlight the ability of these optical nanoprobes to capture the infiltration of MDSCs into the lungs during the development of the PMN in models of high metastatic propensity. While the increased in vivo signal from Gr-ReANCs in the less aggressive EMT6 model is not statistically significant, the marked increases in the ex vivo imaging suggests that this platform may be able to produce smaller increases in MDSC infiltration for more insidious disease progression if imaged at additional timepoints. The results of this study support the ReANC technology as a viable radiation-free methodology for longitudinal monitoring of PMN development in post-operative patients during this critical period. The reduction in the relative Gr-ReANC signal at day 21 compared to Iso-ReANC suggests that MDSCs may only provide a window for detection of the PMN. Beyond this window period, the increased permeability of the vasculature and poor lymphatic drainage of the microenvironment result in the non-specific accumulation of ReANCs, masking the detection of MDSC infiltration [30,50]. Therefore, beyond a certain stage of niche development, targeted nanoprobes may no longer be necessary as the lesion becomes more advanced.

The differences between in vivo and ex vivo imaging results can be explained by the signal attenuation from tissue transversal. While SWIR light is superior to UV and visible wavelengths for tissue penetration, it still experiences a degree of attenuation due to tissue absorption and scattering [27]. This in turn will result in a reduction in the signal-to-noise ratio when imaging through intact tissue. The signal strength and signal-to-noise ratio will always be improved in ex vivo optical imaging. In contrast to the athymic study that had only modest MDSC infiltration in the EMT6 tumor model, the Balb/c mice showed significant MDSC infiltration in both 4T1 and EMT6 tumor models. This could also be attributed to the signal attenuation that may result from the hair of the mice. Ex vivo imaging results of both studies validate our in vivo findings, with elevated Gr-ReANC signal compared to control in only 4T1 mice in the athymic study and relative elevation of Gr-ReANC signal compared to the control in both tumor models in Balb/c mice.

A limitation of this study is that MDSCs may be difficult to characterize by a single marker, as many of the surface markers are common amongst other myeloid lineages [52]. Our previous work demonstrates that ReANCs may be formulated with unique emission wavelengths for the detection of unique molecular markers [30,36]. The use of a second probe will allow for more specific cell detection by imaging multiple markers for the co-registration of multiple markers indicative of MDSCs. In addition, multispectral probes may be utilized for MDSCs to image additional immune subsets, such as cytotoxic T cells, providing further detailed information of the tumor immune environment which may inform on therapy responsiveness and disease progression [39].

MDSCs have been implicated in the resistance of tumors to immunotherapy; however, this technology is a delivery mechanism for targeted therapy to re-sensitize tumors [53,54]. Our lab has previously leveraged the drug binding pockets on the albumin shell of ReANCs to deliver chemotherapy for the theranostic monitoring and treatment of lung metastases [33]. This approach has the potential to be adopted for theranostic monitoring of the PMN. Recent evidence has shown that MDSC depletion with anti-Gr-1 antibodies or gemcitabine may prevent metastasis formation and rescue the immunotherapy response of tumors [19,48,55,56,57]. The coupling of therapies that eliminate MDSCs with Gr-ReANCs will permit a targeted approach to monitoring disease progression through SWIR imaging with simultaneous treatment of potential metastatic sites. The depletion of MDSCs may serve an additional purpose, as the MDSCs may be responsible for the release of cancer cells from dormancy within the PMN. Within the primary tumor or macro-metastases, this approach may sensitize tumors to immune checkpoint inhibitors through the depletion of MDSCs [53,54,55,58]. The dual action of MDSC imaging and depletion could serve as a (neo)adjuvant therapy, allowing clinicians to assess the risk of metastatic recurrence while simultaneously treating existing lesions and potentially reversing the immunosuppression of the PMN and preventing metastasis formation [19]. The theranostic approach is favorable due to the reduction in systemic toxicity associated with targeted delivery of therapeutics [59], and in a more practical sense, a reduction in the total number of individual agents administered to a patient.

In conclusion, these studies have demonstrated the ability of Gr-ReANCs to target MDSCs and track their migration to the lung PMN in a spontaneous metastasis models of breast cancer early in disease progression. The utility of this technology lies not only in the MDSC imaging capabilities but also the multispectral imaging and theranostic approaches afforded by the RENP dopants and albumin shell. Future studies will seek to explore how the multispectral imaging and theranostic capabilities of MDSCs may be leveraged to discern the PMN, tumor immunogenicity, and targeted depletion of MDSCs. The theranostic potential of Gr-ReANCs could aid in the proactive reversal of PMN immunosuppression, preventing micro-metastases from seeding in favorable environments. This will aid in the advancement of precision medicine and the prognostication of disease progression.

## Data Availability

All data are available upon request.

## Supplementary Materials

The following supporting information can be downloaded at https://www.mdpi.com/article/10.3390/pharmaceutics16040549/s1, Figure S1: IgG-ReANC sizing and IgG-ANC MDSC binding assay; Figure S2: Characterization of MDSC lung infiltration; Figure S3: Athymic mouse weights and tumor growth; Figure S4: Balb/c mouse weights and tumor growth; Figure S5: Immunohistochemistry staining of lungs; Figure S6: Individual study results from 4T1 mice; Figure S7: Individual study results from EMT6 tumor bearing mice; Figure S8: SWIR images of athymic mice.
