# Supplementary material for: Early Detection of Myeloid-Derived Suppressor Cells in the Lung Pre-Metastatic Niche by Shortwave Infrared Nanoprobes
Source: Pharmaceutics. 2024 Apr 17;16(4):549. doi: 10.3390/pharmaceutics16040549 (PMC11053826; doi:10.3390/pharmaceutics16040549)
Supplement: Supplementary file 1 [file pharmaceutics-16-00549-s001.zip › pharmaceutics-2919667-supplementary.pdf]

pharmaceutics

MDPI

Supplementary Materials: Early Detection of Myeloid-Derived Suppressor Cells in the Lung Pre-Metastatic Niche by Shortwave Infrared Nanoprobes

Jake N. Siebert, Jay V. Shah, Mei Chee Tan, Richard E. Riman, Mark C. Pierce, Edmund C. Lattime, Vidya Ganapathy and Prabhas V. Moghe

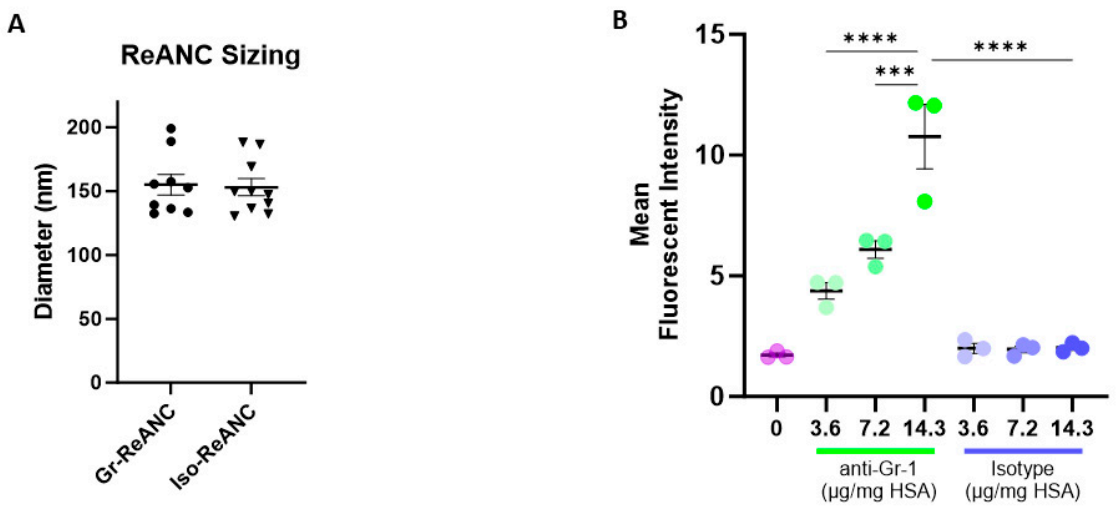

Figure S1. IgG-ReANC Sizing and IgG-ANC MDSC Binding Assay. **A)** The hydrodynamic diameters of Gr-ReANCs and Iso-ReANCs were determined by dynamic light scattering (DLS). The average hydrodynamic diameter was 155.0 ± 8.0 nm and 153.0 ± 6.7 nm for Gr-ReANCs (PDI: 0.17) and Iso-ReANCs (PDI: 0.15). **B)** MDSC binding of Gr-ANCs and Iso-ANCs assessed by flow cytometry with IgG loading concentrations of 3.6 to 14.3 μg mg^{-1} HSA.

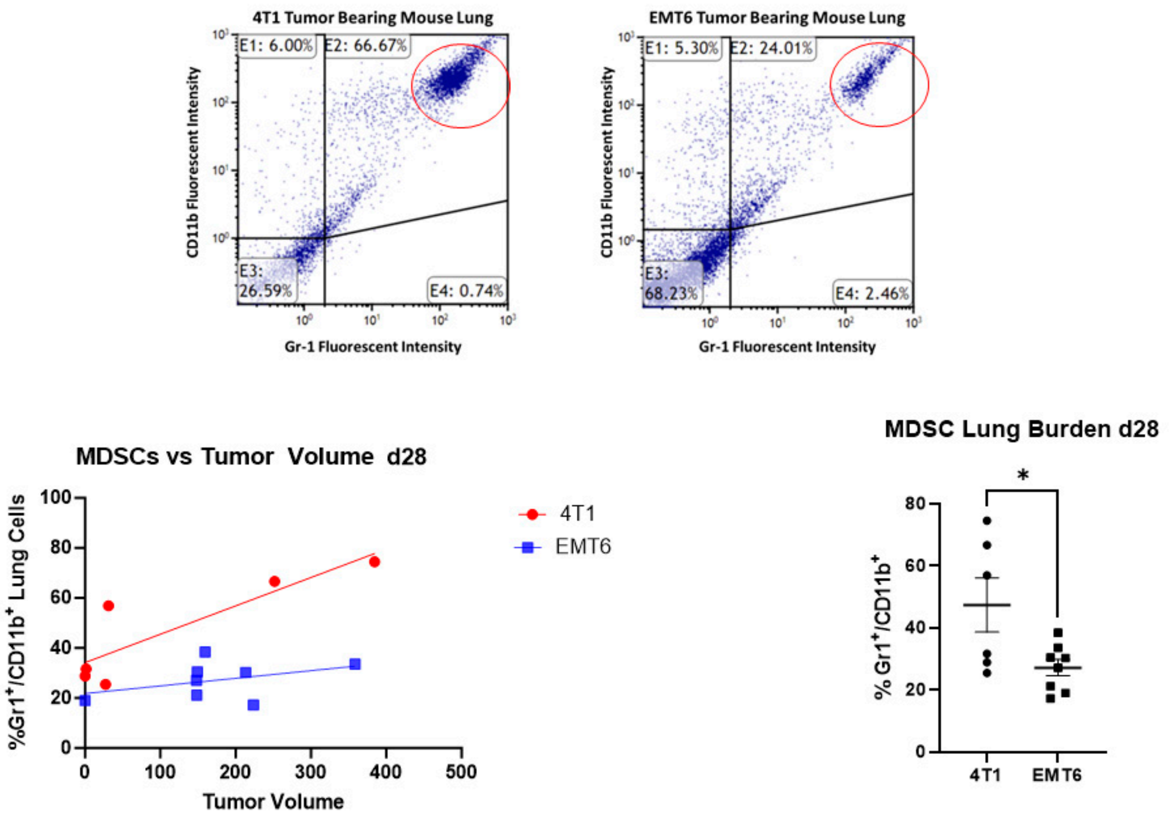

Figure S2. Characterization of MDSC lung infiltration in two murine syngeneic mammary fat pad models of breast cancer, 4T1 and EMT6, with spontaneous lung metastasis. **Top:** Flow cytometry gating of single cell lung suspensions stained for Gr-1$^+$ and CD11b$^+$ cells in both 4T1 and EMT6 mice. 66.7% of cells stained positive for both markers in 4T1 mice compared to EMT6 mice. **Bottom:** Flow cytometry results on day 28 post-inoculation demonstrates significantly increased MDSC lung infiltration in 4T1 mice.

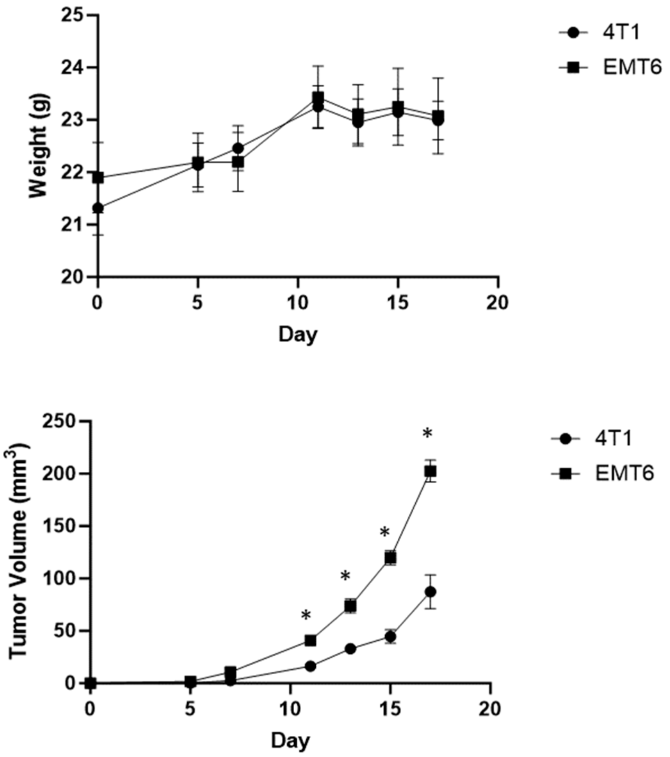

Figure S3. Athymic mouse weights and tumor growth. **Top:** Mouse weights for EMT6 and 4T1 tumor bearing athymic nude mice from day 0 to day 17. **Bottom:** 4T1 and EMT6 tumor volumes, EMT6 tumors are significantly larger than 4T1 tumors from day 11 on ($p<0.0001$).

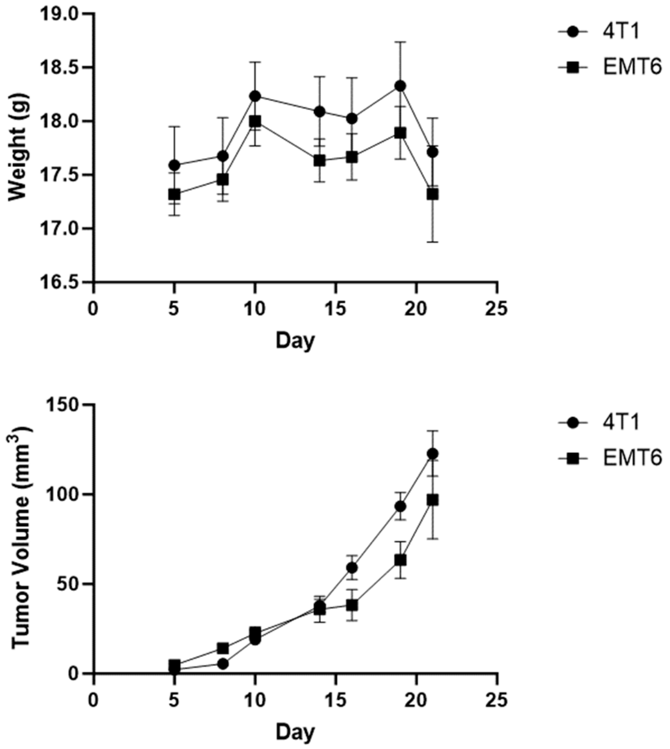

Figure S4. Balb/c mouse weights and tumor growth. **Top:** Average mouse weights during the course of study 3 in Balb/c mice. **Bottom:** Average volume of 4T1 and EMT6 tumors during study 3, no difference in tumor volume on day 14 (p=0.799) or day 21 (p=0.327).

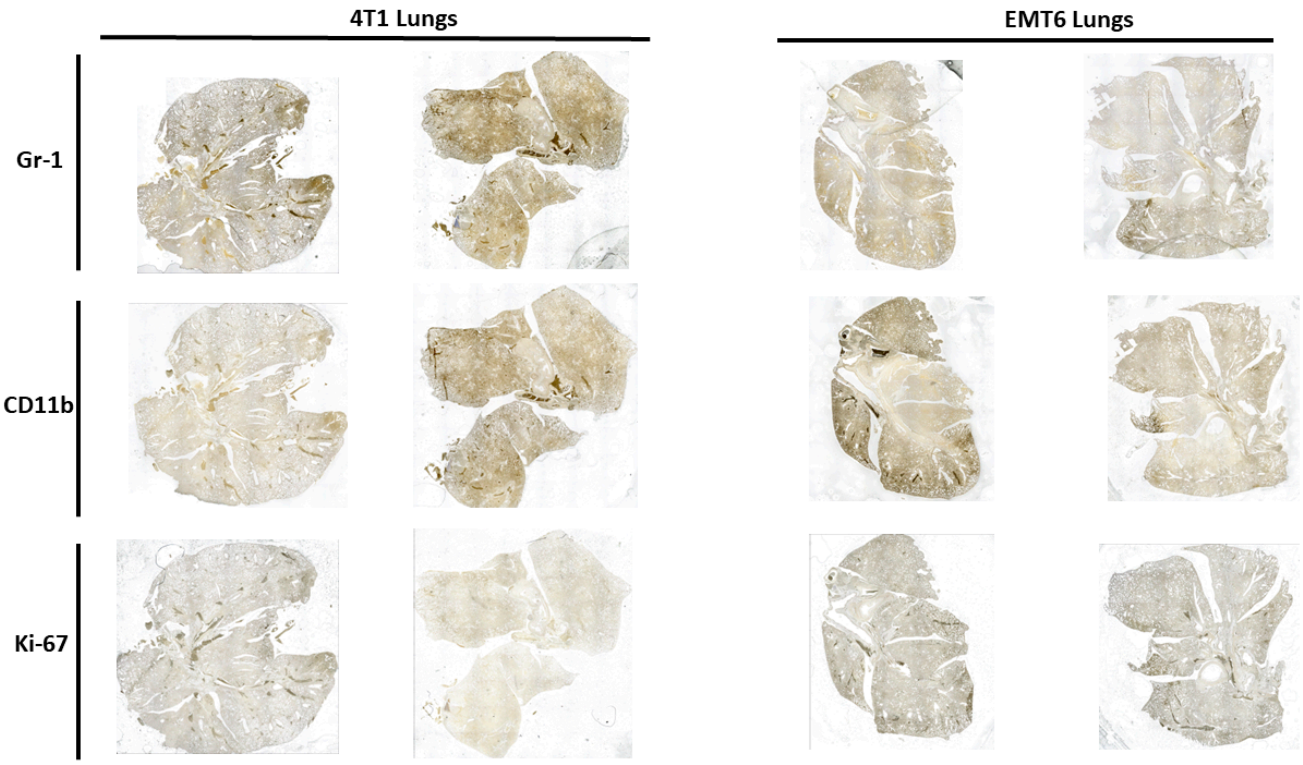

Figure S5. Representative lung samples from 4T1 and EMT6 tumor bearing mice stained for MDSCs, Gr-1 and CD11b, and proliferation marker Ki-67. Positive staining appears brown.

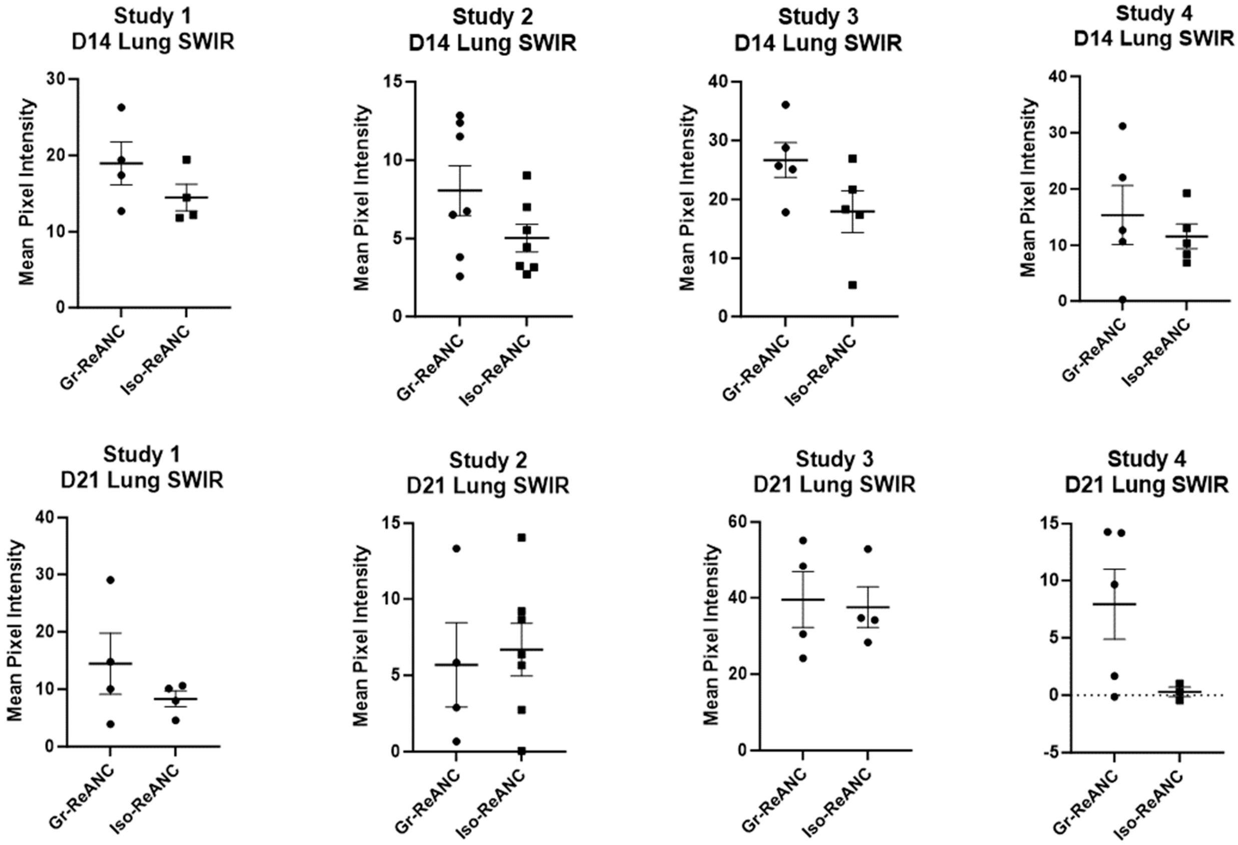

Figure S6. Individual study results from 4T1 mice on day 14 (top) and day 21 (bottom). Individual study results demonstrate a consistent increase in SWIR signal with Gr-ReANCs compared to Iso-ReANCs on day 14 across all studies. Day 21 results demonstrate variability in differences between Gr-ReANC and Iso-ReANC.

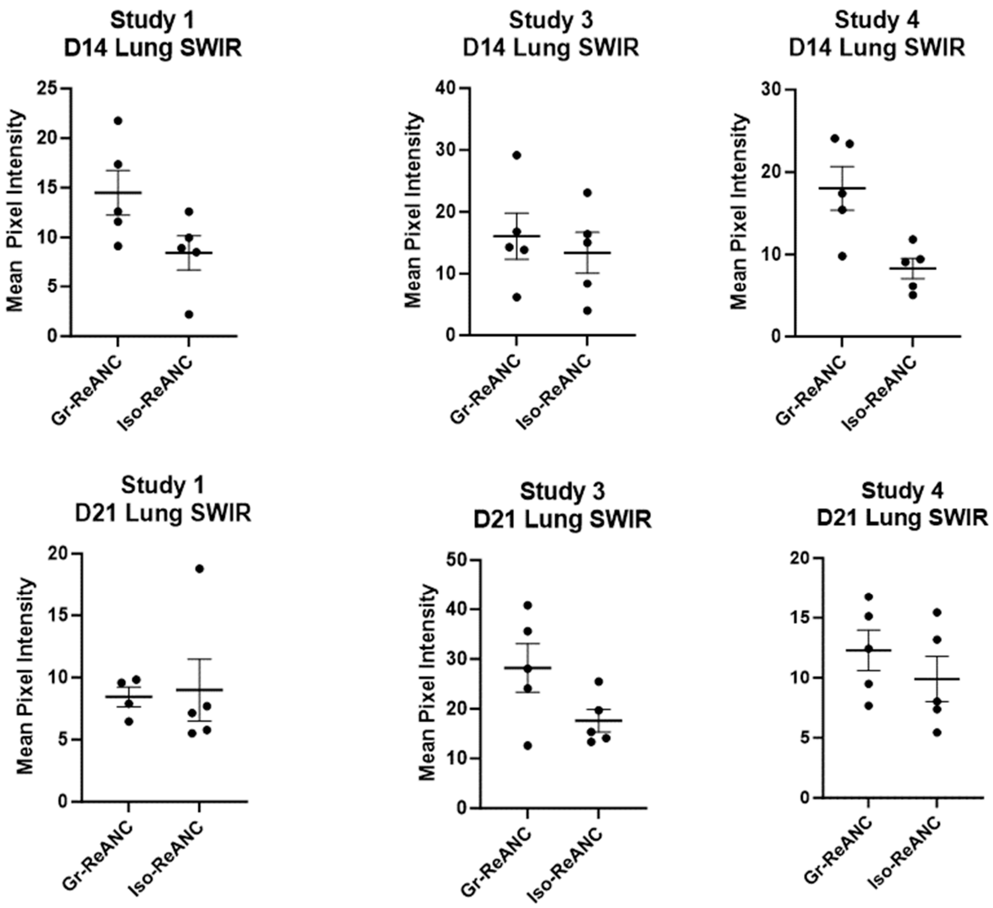

Figure S7. Individual study results from EMT6 tumor bearing mice on day 14 (top) and day 21 (bottom). Results show variability in relative differences between Gr-ReANC SWIR signal and Iso-ReANCs at both day 14 and day 21.

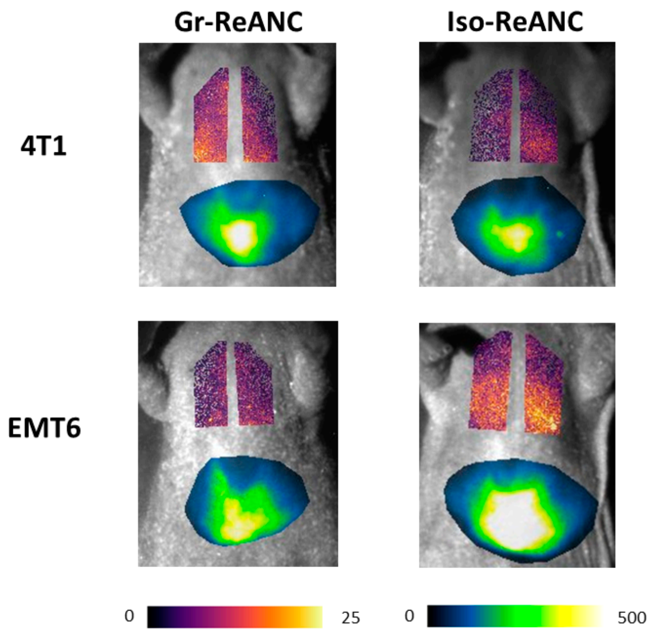

Figure S8. False cover SWIR images of athymic mice with 4T1 or EMT6 tumors treated with either Gr-ReANCs or Iso-ReANCs, corresponding to the quantified pixel intensity data from Figure 3A. The results show increased SWIR intensity in 4T1 tumor bearing mice treated with Gr-ReANCs compared to Iso-ReANCs and EMT6 tumor bearing mice treated with Gr-ReANCs. There is no difference between EMT6 mice treated with Gr-ReANCs and Iso-ReANCs.
